# Obatoclax, a Pan-BCL-2 Inhibitor, Targets Cyclin D1 for Degradation to Induce Antiproliferation in Human Colorectal Carcinoma Cells

**DOI:** 10.3390/ijms18010044

**Published:** 2016-12-27

**Authors:** Chi-Hung R. Or, Yachu Chang, Wei-Cheng Lin, Wee-Chyan Lee, Hong-Lin Su, Muk-Wing Cheung, Chang-Po Huang, Cheesang Ho, Chia-Che Chang

**Affiliations:** 1 Institute of Biomedical Sciences, National Chung Hsing University, 145 Xingda Road, Taichung 40227, Taiwan; yachu_0712@hotmail.com (Y.C.); pa6153680@gmail.com (W.-C.Li.); chyan_9@hotmail.com (W.-C.Le.); 2 Department of Life Science, National Chung Hsing University, 145 Xingda Road, Taichung 40227, Taiwan; richardor92@yahoo.com.tw (C.-H.R.O.); suhonglin@gmail.com (H.-L.S.); 3 Department of Anesthesiology, Kuang Tien General Hospital, Dajia Branch, 321 Jingguo Road, Taichung 43761, Taiwan; garrettc1957@yahoo.com.tw (M.-W.C.); vincent520330@yahoo.com.tw (C.-P.H.); b611071@tmu.edu.tw (C.H.); 4 Ph.D. Program in Translational Medicine, National Chung Hsing University, 145 Xingda Road, Taichung 40227, Taiwan; 5 Agricultural Biotechnology Center, National Chung Hsing University, 145 Xingda Road, Taichung 40227, Taiwan; 6 Rong Hsing Research Center for Translational Medicine, National Chung Hsing University, 145 Xingda Road, Taichung 40227, Taiwan; 7 Department of Medical Research, China Medical University Hospital, 2 Yude Road, Taichung 40447, Taiwan; 8 Department of Biotechnology, Asia University, 500 Liufeng Road, Taichung 41354, Taiwan

**Keywords:** obatoclax, BH3 (BCL-2 homology 3) mimetics, cyclin D1, proteasomal degradation, G_1_-phase arrest, antiproliferation, colorectal cancer

## Abstract

Colorectal cancer is the third most common cancer worldwide. Aberrant overexpression of antiapoptotic BCL-2 (B-cell lymphoma 2) family proteins is closely linked to tumorigenesis and poor prognosis in colorectal cancer. Obatoclax is an inhibitor targeting all antiapoptotic BCL-2 proteins. A previous study has described the antiproliferative action of obatoclax in one human colorectal cancer cell line without elucidating the underlying mechanisms. We herein reported that, in a panel of human colorectal cancer cell lines, obatoclax inhibits cell proliferation, suppresses clonogenicity, and induces G_1_-phase cell cycle arrest, along with cyclin D1 downregulation. Notably, ectopic cyclin D1 overexpression abrogated clonogenicity suppression but also G_1_-phase arrest elicited by obatoclax. Mechanistically, pre-treatment with the proteasome inhibitor MG-132 restored cyclin D1 levels in all obatoclax-treated cell lines. Cycloheximide chase analyses further revealed an evident reduction in the half-life of cyclin D1 protein by obatoclax, confirming that obatoclax downregulates cyclin D1 through induction of cyclin D1 proteasomal degradation. Lastly, threonine 286 phosphorylation of cyclin D1, which is essential for initiating cyclin D1 proteasomal degradation, was induced by obatoclax in one cell line but not others. Collectively, we reveal a novel anticancer mechanism of obatoclax by validating that obatoclax targets cyclin D1 for proteasomal degradation to downregulate cyclin D1 for inducing antiproliferation.

## 1. Introduction

Colorectal cancer was the third most commonly diagnosed cancer and the fourth leading cause of cancer-related death globally in 2012 [1]. Accumulation of inactivating mutations in tumor suppressor genes such as *adenomatous polyposis coli* (*APC*), *SMAD4*, or *TP53* and oncogenic mutations in *KRAS*, *PI3KCA*, or *BRAF* are critical for the development and progression of colorectal cancer. In particular, loss of APC and consequent increase in β-catenin facilitates the formation of adenoma, thereby initiating the adenoma-carcinoma sequence for colorectal tumorigenesis [2]. Colorectal cancer is often first diagnosed at an advanced stage [3]. Surgical resection followed by systemic chemotherapy is recommended for the treatment of patients with advanced colorectal cancer; however, clinical relapse frequently occurs and accounts for most colorectal cancer-related mortality [4]. Identification of novel chemotherapeutics with better efficacy for colorectal cancer therapy is therefore in urgent demand.

Apoptosis constitutes a fundamental intrinsic mechanism of tumor suppression, as the resistance of apoptosis is a well-established hallmark of cancer [5]. Apoptosis is predominantly regulated by members of the BCL-2 (B-cell lymphoma 2) family [6]. The antiapoptotic members, including BCL-2, BCL-xL (B-cell lymphoma-extra large), and MCL-1 (Myeloid cell leukemia 1), suppress apoptosis by binding to proapoptotic BAK (BCL-2 homologous antagonist/killer) and BAX (BCL-2-associated X protein) to prevent their activation for initiating the mitochondrial apoptotic signaling. Furthermore, proapoptotic BH3-only proteins function either as BAX/BAK activators through direct binding to BAX/BAK, or act as sensitizers of proapoptotic stimuli by fitting into the BH3-binding groove of antiapoptotic members to release the activator BH3-only proteins for BAX/BAK activation [7]. As to colorectal cancer, it has been proven that aberrant overexpression of antiapoptotic BCL-2 family proteins is closely linked to colorectal tumorigenesis, poor prognosis, and drug resistance, thus highlighting the potential of drugs targeting antiapoptotic BCL-2 proteins for colorectal cancer therapy [8,9,10]. To this end, a novel class of cancer therapeutics coined as “BH3 mimetics”, including ABT-737, ABT-263, and obatoclax, were developed to function as inhibitors of antiapoptotic BCL-2 members by mimicking the modes of action of BH3-only proteins [11,12,13,14,15]. These BH3 mimetics are potent inducers of apoptosis in vitro and have been under intensive clinical trials [11,15].

Obatoclax is classified as a BH3 mimetic by its ability to bind to the BH3-bidning groove of BCL-2, BCL-xL, and MCL-1, leading to inhibition of these antiapoptotic BCL-2 proteins and consequent BAX/BAK-dependent apoptosis [11,16,17]. Obatoclax as a single agent induces apoptosis in cells derived from hematological cancers and solid tumors, but also potentiates the cytotoxicity of conventional chemotherapeutics or targeted therapy drugs [17,18,19]. Of note, obatoclax is unique by its inhibitory action on MCL-1 compared to ABT-737 and its orally available derivative ABT-263 (navitoclax), which bind to BCL-2 and BCL-xL, but not MCL-1, thus allowing obatoclax to overcome MCL-1-mediated ABT-737 resistance [20]. Phase III clinical trials of obatoclax in combination therapies are currently ongoing [11,15].

Intriguingly, evidence that obatoclax can also evoke BAX- and BAK-independent cell death [17] highlights additional mechanisms of action that account for the anticancer effect of obatoclax. Indeed, obatoclax has been documented to induce autophagic cell death [21], likely due to blocking the end stage of autophagy through inhibition of lysosomal activity [22,23]. Furthermore, the obatoclax-mediated death of rhabdomyosarcoma cells is attributed to the induction of necroptosis [24]. It is also noteworthy that, aside from cell death, inhibition of cell proliferation by delaying cell cycle progression at G_1_- or S-phase has also been reported for the anticancer action of obatoclax [17,25,26].

Previous studies by Koheler et al. [26] have explored the antiproliferative action of obatoclax by showing the ability of obatoclax to delay G_1_-phase cell cycle progression and lower the levels of cyclin D1 in human colorectal cell line HT-29 [26]. However, in Koheler’s report obatoclax-induced antiproliferation and G_1_-phase arrest were only demonstrated in a single cell line HT-29; furthermore, no direct evidence was provided to support an increase in the G_1_-phase cell population. Moreover, it remains unknown as to the role of cyclin D1 downregulation in obatoclax-mediated G_1_-phase arrest and antiproliferation, nor the mechanisms of action underlying obatoclax-elicited cyclin D1 downregulation. To address these unresolved questions, we herein provide the first evidence to prove that, in a panel of human colorectal cancer cell lines, obatoclax inhibits cell proliferation and provokes G_1_-phase arrest as a result of cyclin D1 downregulation through accelerating proteasome-mediated cyclin D1 degradation. Our findings, therefore, support antiproliferation induction as a general mechanism underlying obatoclax-mediated anticancer action, but also identify obatoclax as a therapeutic cyclin D1-ablating agent that targets cyclin D1 for proteasome-mediated degradation.

## 2. Results

### 2.1. Obatoclax Inhibits Cell Proliferation and Induces G_1_ Cell-Cycle Arrest in a Panel of Human Colorectal Cancer Cell Lines

**Figure 1:**
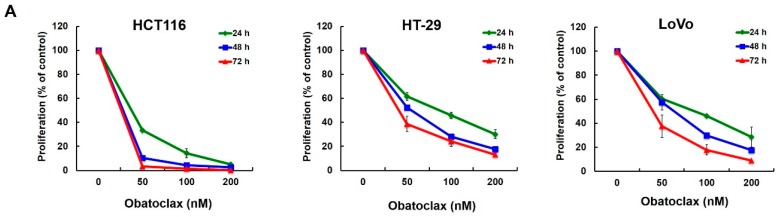

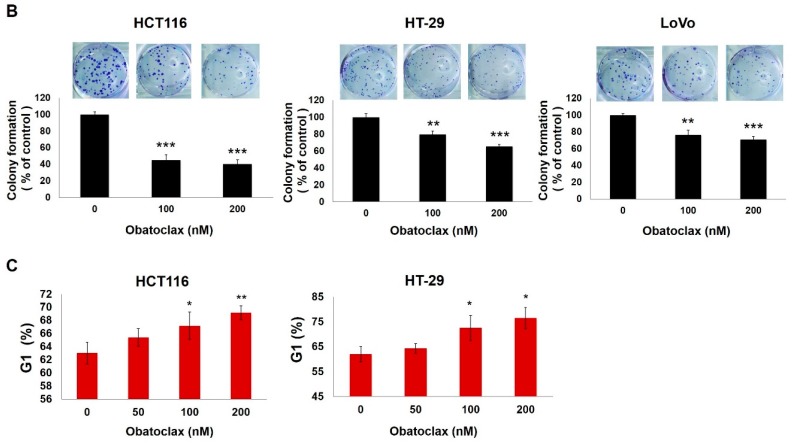
Antiproliferative action of obatoclax on a panel of human colorectal cancer cell lines. (**A**) Obatoclax inhibits colorectal cancer cell proliferation. Human colorectal carcinoma cell lines HCT116, HT-29, and LoVo cells (4 × 10^4^) were treated with obatoclax (0, 50, 100, 200 nM) for 24, 48, and 72 h. The numbers of cells at each time point were counted using a Neubauer chamber. Data were presented as the percentage relative to the drug-untreated control; (**B**) Obatoclax suppresses colony formation by colorectal cancer cells. (**Upper panel**) HCT116, HT-29, and LoVo cells (2 × 10^2^) treated with obatoclax (0, 100, 200 nM) for 24 h were allowed to proliferate in drug-free culture media for 10~14 days to form colonies, followed by crystal violet staining for scoring colonies. Shown here are the representative images from three independent experiments. (**Lower panel**) Quantitative results of the upper panels. Data were presented as the percentage relative to the drug-untreated control; (**C**) Obatoclax arrests cell cycle progression at the G_1_-phase. HCT116 and HT-29 cells were treated with obatoclax (0, 50, 100, 200 nM) for 24 h, followed by flow cytometry analyses to determine the levels of cell population at the G_1_-phase of the cell cycle. Data were presented as the percentage relative to the drug-untreated control. *: *p* < 0.05; **: *p* < 0.01; ***: *p* < 0.001.

To examine obatoclax-induced antiproliferation, we treated human colorectal cancer cells with grading doses of obatoclax (0~200 nM), followed by counting cell numbers 24, 48, and 72 h after drug stimulation. Three distinct human colorectal cancer cell lines, including HCT116, HT-29, and LoVo, were examined for obatoclax-induced antiproliferation in order to exclude the possibility of a cell-specific effect of obatoclax. As shown in Figure 1A, obatoclax induced a dose- and time-dependent reduction of cell numbers in all tested cell lines. In particular, the doses of obatoclax required to suppress 50% (IC_50_) of cell proliferation at 72 h were 25.85, 40.69, and 40.01 nM for HCT116, HT-29, and LoVo cells, respectively. We next addressed whether obatoclax is capable of inhibiting the colony-forming capacity of human colorectal cancer cells. Our results showed that obatoclax markedly suppressed the clonogenicity of all tested cell lines (Figure 1B), hence confirming the action of obatoclax to block long-term cell proliferation. Moreover, cell-cycle progression analyses revealed that obatoclax provoked a dose-dependent increase in the G_1_-phase cell populations in both HCT116 and HT-29 cells (Figure 1C), illustrating G_1_-phase arrest of the cell cycle. Overall, these findings support that induction of antiproliferation by arresting cell cycle progression at the G_1_-phase is likely a universal action of mechanism underlying the anti-colorectal cancer effect of obatoclax.

### 2.2. Cyclin D1 Downregulation Is Fundamental for Obatoclax-Induced Antiproliferation

**Figure 2:**
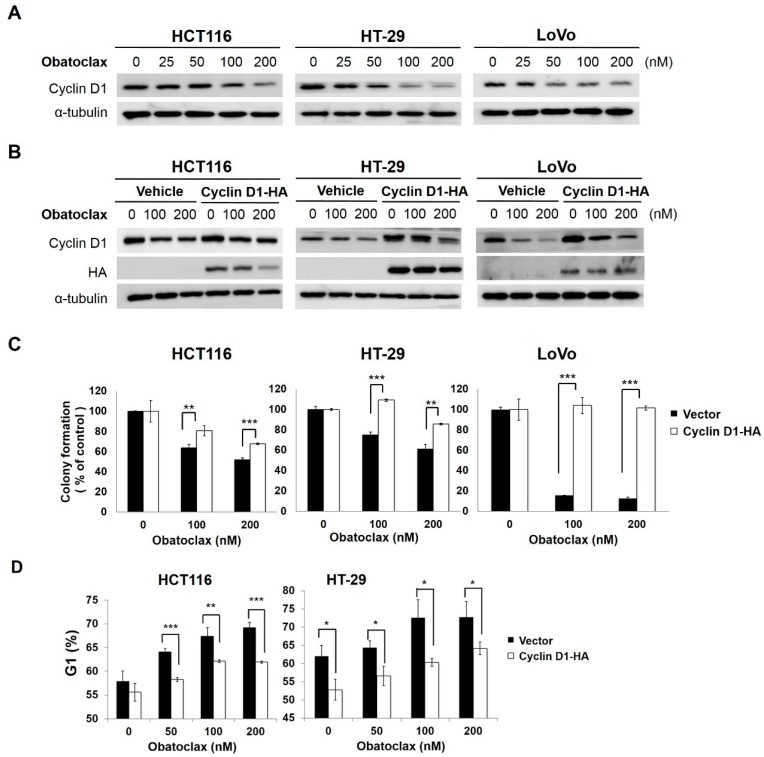
Obatoclax downregulates cyclin D1 to induce G_1_-phase arrest and consequent antiproliferation. (**A**) Obatoclax downregulates cyclin D1. HCT116, HT-29, and LoVo cells were exposed to obatoclax (0, 25, 50, 100, 200 nM) for 24 h, followed by cyclin D1 immunoblotting. α-Tubulin levels served as the control for equal loading; (**B**) Overexpression of cyclin D1 attenuates obatoclax-elicited reduction of cyclin D1. Stable clones of HCT116, HT-29, and LoVo cells with ectopic expression of hemagglutinin (HA)-tagged cyclin D1 were treated with obatoclax (0, 100, 200 nM) for 24 h. HA immunoblotting was performed to confirm ectopic expression of HA-tagged cyclin D1, and cyclin D1 immunoblotting was used to verify the resistance to the obatoclax-induced decrease in cyclin D1 levels. α-Tubulin levels served as the control for equal loading; (**C**) Overexpression of cyclin D1 rescues colony formation capability of obatoclax-treated cells. HA-Cyclin D1 stable clones and their corresponding vector controls were treated with obatoclax (0, 100, 200 nM) for 24 h, followed by colony formation assays. Significant restoration of colony numbers was observed in the group with overexpression of HA-Cyclin D1. Data were presented as the percentage relative to the drug-untreated control; (**D**) Overexpression of cyclin D1 abrogates obatoclax-elicited G1-phase arrest. HA-Cyclin D1 stable clones and their corresponding vector controls were treated with obatoclax (0, 50, 100, 200 nM) for 24 h, followed by cell cycle analysis to determine the levels of cell population at the G_1_-phase. A marked reduction of cells at the G_1_ stage was revealed in HA-Cyclin D1 stable clones treated with obatoclax. Data were presented as the percentage relative to the drug-untreated control. *: *p* < 0.05; **: *p* < 0.01; ***: *p* < 0.001.

How obatoclax induces G_1_-phase arrest was next investigated. It is well-established that cyclin D1 is essential for driving G_1_-to-S transition to promote cell cycle progression. Importantly, cyclin D1 overexpression leads to uncontrolled cell proliferation and is linked to the development, progression, poor prognosis, and chemoresistance of a broad range of human malignancies, including colorectal cancer, thus making cyclin D1 a potential therapeutic target [27,28]. Accordingly, we examined the effect of obatoclax on cyclin D1 expression in HCT116, HT-29, and LoVo cells. Our results indicated a marked drop in cyclin D1 levels following treatment with obatoclax as low as 50 nM (Figure 2A). To further substantiate the functional significance of cyclin D1 downregulation in obatoclax-induced antiproliferation, we established stable clones overexpressing HA (Hemagglutinin)-tagged cyclin D1 to counteract obatoclax-induced reduction of cyclin D1 (Figure 2B). It is noteworthy that in all cell lines tested, ectopic cyclin D1 overexpression rescued the clonogenicity of obatoclax-treated cells (Figure 2C), but also attenuated the levels of obatoclax-elicited G_1_-phase arrest (Figure 2D). In sum, we conclude that cyclin D1 downregulation appears as a primary mode of action whereby obatoclax provokes G_1_-phase cell cycle arrest, leading to antiproliferation of colorectal cancer cells.

### 2.3. Obatoclax Downregulates Cyclin D1 Primarily through Inducing Cyclin D1 Protein Degradation

**Figure 3:**
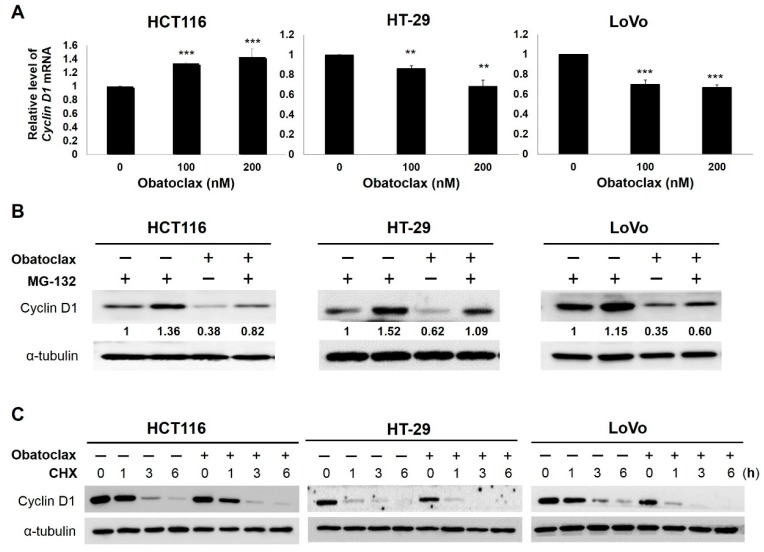

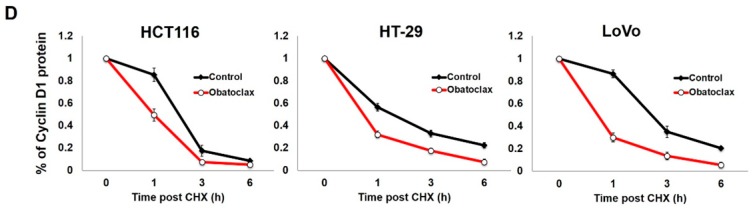
Obatoclax targets cyclin D1 for proteasome-mediated degradation. (**A**) Cell line-dependence of the effect of obatoclax on cyclin D1 mRNA expression. HCT116, HT-29, and LoVo cells were stimulated by obatoclax (0, 100, 200 nM) for 24 h, followed by determination of *cyclin D1* mRNA levels using quantitative real-time reverse transcription-polymerase chain reaction (RT-PCR) analysis. Data were presented as the percentage relative to the drug-untreated control. **: *p* < 0.01. ***: *p* < 0.001; (**B**) Blockade of proteasome-mediated degradation restores cyclin D1 levels downregulated by obatoclax. HCT116, HT-29, and LoVo cells were treated with obatoclax (0, 100, 200 nM) for 24 h. The proteasome inhibitor MG-132 was then added to obatoclax-treating cells and the MG-132 co-treatment was allowed to occur for 2 h, followed by cyclin D1 immunoblotting. It is noticed that cyclin D1 levels were significantly rescued by the MG-132 co-treatment. α-Tubulin levels served as the control for equal loading; (**C**) Obatoclax destabilizes cyclin D1. HCT116, HT-29, and LoVo cells were treated with obatoclax (200 nM) for 24 h and then subject to cycloheximide chase analysis. The levels of cyclin D1 at 0, 1, 3, and 6 h after cycloheximide treatment were determined using immunoblot analysis. α-Tubulin levels served as the control for equal loading; (**D**) Obatoclax elevates the rate of cyclin D1 protein degradation. The signal density of each band in the blots shown in (**C**) was quantified using ImageJ 1.48s algorithms and then plotted against the length of time after cycloheximide treatment. Data were presented as the percentage relative to the drug-untreated control. Data indicated that the half-life of cyclin D1 protein was dropped from 1.48 to 0.49 h in HCT116 cells, from 1.55 to 0.74 h in HT-29 cells, and from 2.42 to 0.71 h in LoVo cells.

We next explored how obatoclax downregulates cyclin D1, whose levels are mainly subject to transcriptional and/or post-translational control [29,30]. With respect to transcriptional control, we found that *cyclin D1* mRNA levels were significantly elevated in HCT116 cells while reduced in HT-29 and LoVo cells after obatoclax treatment (Figure 3A), therefore excluding transcriptional control as a general mechanism whereby obatoclax downregulates cyclin D1. To assess the role of post-translational regulation, cells were treated with obatoclax (200 nM) without or with co-treatment of MG-132 to block proteasome-mediated degradation, followed by cyclin D1 immunoblotting. Of note, in all tested cell lines, MG-132 co-treatment effectively rescued cyclin D1 expression to the levels comparable to those of drug-free controls (Figure 3B), suggesting that obatoclax generally targets cyclin D1 for proteasomal degradation to lower cyclin D1 abundance. To further substantiate obatoclax-induced cyclin D1 degradation, we conducted cycloheximide chase analyses to evaluate the effect of obatoclax on the stability of cyclin D1 protein. Our data clearly proved a marked increase in the rate of degradation of cyclin D1 protein in obatoclax-treated cells (Figure 3C), confirming destabilization of cyclin D1 protein elicited by obatoclax. Specifically, the half-life of cyclin D1 protein was dropped from 1.48 to 0.49 h in HCT116 cells, from 1.55 to 0.74 h in HT-29 cells, and from 2.42 to 0.71 h in LoVo cells (Figure 3D). Altogether, these results support that obatoclax-induced cyclin D1 downregulation is mainly achieved by targeting cyclin D1 for proteasomal degradation.

### 2.4. Obatoclax Induces Cyclin D1 Degradation in a T286 Phosphorylation-Dependent or -Independent Manners

**Figure 4:**
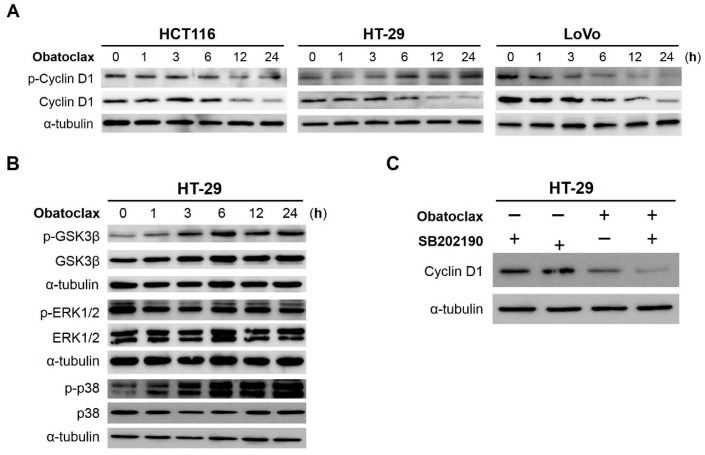
Obatoclax induces T286 phosphorylation-dependent or -independent cyclin D1 degradation. (**A**) Cell line-dependent effect of obatoclax on cyclin D1 T286 phosphorylation. HCT116, HT-29, and LoVo cells were subject to 24 h-treatment with obatoclax (200 nM), followed by immunoblotting to evaluate the levels of threonine 286 (T286)-phosphorylated cyclin D1 (p-Cyclin D1) at indicated time points. α-Tubulin levels served as equal loading control; (**B**) Obatoclax inhibits GSK3β but activates p38^MAPK^, while barely affecting ERK1/2 activity in HT-29 cells. HT-29 cells were exposed to 200 nM of obatoclax for 24 h. The status of activation of GSK3β, ERK1/2, and p38^MAPK^ at indicated time points was determined by immunoblotting for corresponding phosphorylation. Specifically, serine 9-phosphorylation of GSK3β (p-GSK3β) reveals inhibition of GSK3β activity, whereas dual phosphorylation at threonine 202/tyrosine 204 of ERK1/2 (p-ERK1/2) and threonine 180/tyrosine 182 of p38^MAPK^ (p-p38) indicate activation of ERK1/2 and p38^MAPK^, respectively. α-Tubulin levels served as equal loading control; (**C**) p38^MAPK^ activity is not required for obatoclax-induced cyclin D1 downregulation in HT-29 cells. HT-29 cells were exposed to obatoclax without or with pre-treatment of the p38^MAPK^-specific inhibitor SB202190 (10 µM) for 24 h, followed by cyclin D1 immunoblotting. The data revealed that blockade of p38^MAPK^ activity fails to restore cyclin D1 levels downregulated by obatoclax.

How obatoclax accelerates cyclin D1 degradation was further examined. In general, cyclin D1 degradation is initiated by phosphorylation at threonine 286 (T286), followed by ubiquitination and proteasomal degradation [30]. In view of that, we monitored the levels of T286-phosphorylated cyclin D1 (p-Cyclin D (T286)) during 24 h-treatment with obatoclax. Surprisingly, we found that, in HCT116 and LoVo cells, the steady-state levels of p-Cyclin D (T286) began to decline once exposed to obatoclax (Figure 4A, left and right panels), thus arguing a mechanism independent of T286 phosphorylation to initiate cyclin D1 degradation in these cells. For HT-29 cells, however, p-Cyclin D (T286) was induced starting at 6 h after obatoclax stimulation, along with a paralleled decrease in cyclin D1 levels (Figure 4A, central panel), suggesting the involvement of T286 phosphorylation-dependent cyclin D1 degradation in obatoclax-treated HT-29 cells.

### 2.5. GSK3β (Glycogen Synthase Kinase 3β), ERK1/2 (Extracellular Signal–Regulated Kinases 1/2), and p38^MAPK^ (p38^Mitogen-Activated Protein Kinase^) Are Dispensable for Obatoclax-Induced Cyclin D1 Degradation in HT-29 Cells

To determine the upstream signaling pathways initiating cyclin D1 degradation in obatoclax-treated HT-29 cells, we asked whether obatoclax induces the activation of kinases known to mediate cyclin D1 T286 phosphorylation, including GSK3β [31], ERK1/2 [32,33], and p38^MAPK^ [34]. We found that obatoclax provoked a time-dependent increase of AKT-mediated GSK3β serine 9 phosphorylation (p-GSK3β (S9)), indicating the inhibition of GSK3β by obatoclax (Figure 4B, upper three blots). Instead, a limited effect of obatoclax on ERK1/2 activation was observed, as the levels of dual phosphorylation on threonine 202 and tyrosine 204 of ERK1/2 (p-ERK1/2 (T202/Y204)) were barely changed by obatoclax (Figure 4B, middle three blots). In contrast, obatoclax clearly promoted the activation of p38^MAPK^, as evidenced by a time-dependent increase of p-p38^MAPK^ (T180/Y182) levels upon obatoclax stimulation (Figure 4B, lower three blots). Nevertheless, pre-treatment with SB202190, a p38^MAPK^-specific inhibitor, failed to restore cyclin D1 levels in obatoclax-treated HT-29 cells (Figure 4C). Collectively, these findings reveal a dispensable role for GSK3β, ERK1/2, and p38^MAPK^ in obatoclax-induced cyclin D1 degradation.

## 3. Discussion

**Figure 5:**
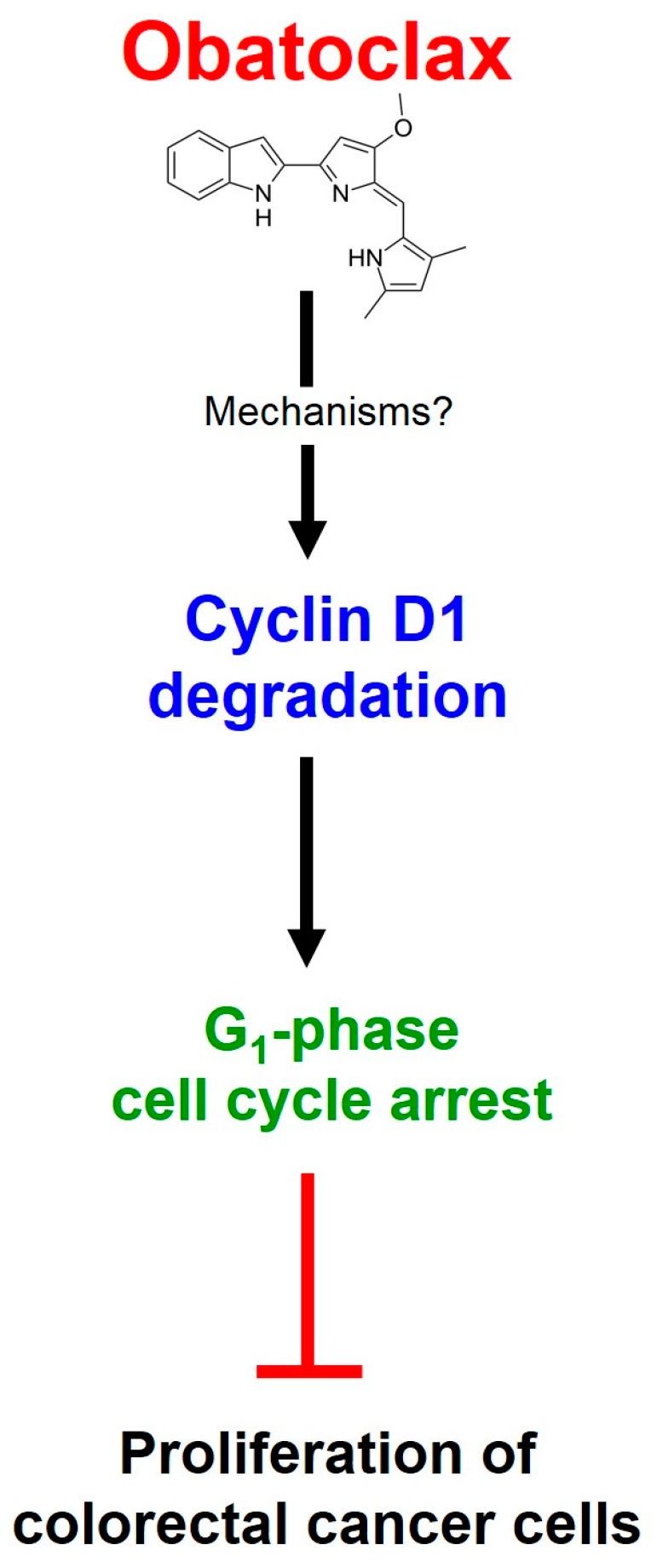
Schematic model depicting the mechanisms of action underlying obatoclax-induced antiproliferation. Obatoclax targets cyclin D1 for proteasome-mediated degradation to downregulate cyclin D1, leading to delayed G_1_-phase cell cycle progression, and the consequent inhibition of cell proliferation in a panel of human colorectal cancer cell lines.

Using a panel of human colorectal cancer cell lines as the cell model, in this study we comprehensively elucidated an obatoclax-induced antiproliferative effect along with the underlying mechanisms. In detail, we demonstrated that obatoclax suppressed cell proliferation, colony-forming capacity, and G_1_-to-S phase transition (Figure 1). Furthermore, the essential role of cyclin D1 downregulation in obatoclax-elicited G_1_ arrest was confirmed (Figure 2). Moreover, induction of cyclin D1 proteasomal degradation was identified as the pivotal mechanism of action for obatoclax to downregulate cyclin D1 (Figure 3). Mechanistically, we revealed that obatoclax provoked cyclin D1 degradation through T286 phosphorylation-dependent or -independent mechanisms, depending on the nature of cell lines (Figure 4). To the best of our knowledge, the notion that obatoclax promotes cyclin D1 degradation to elicit G_1_ cell-cycle arrest for induction of antiproliferation (Figure 5) has never been reported previously.

During our investigation into the anti-colorectal cancer activity of obatoclax, Koheler et al. [26] published a report showing that obatoclax at subtoxic dosage caused G_1_-phase arrest accompanied by cyclin D1 downregulation and delayed cell proliferation in HT-29 cells [26]. In that report, however, obatoclax-induced antiproliferation was only illustrated in a single cell line, HT-29. Furthermore, there was no direct evidence supporting G_1_ arrest but instead it was inferred from reduced cell populations in the G_2_-phase. Additionally, although Koheler et al. [26] showed downregulation of cyclin D1 by obatoclax, the roles of cyclin D1 downregulation in obatoclax-induced G_1_-phase arrest and antiproliferation were not validated, nor were the mechanisms whereby obatoclax downregulates cyclin D1 elucidated. All of these open questions were clearly answered in our study: we validated that obatoclax inhibits cell proliferation by inducing G_1_-phase arrest through accelerating cyclin D1 proteasomal degradation in HCT116 and LoVo cell lines in addition to HT-29 cells (Figure 1, Figure 2 and Figure 3). It is worth noting that HCT116, HT-29, and LoVo cells are genetically heterogeneous with respective to the mutation status of genes involved in colorectal tumorigenesis, including *APC*, *TP53*, *KRAS*, *BRAF*, and *PI3KCA* [35,36,37,38]. Thus, our findings confirm that, regardless of the nature of cell lines, induction of antiproliferation by promoting cyclin D1 proteasomal degradation is a universal mechanism or action underlying obatoclax-mediated anti-colon cancer activity.

Data presented here established the essential role for cyclin D1 downregulation in obatoclax-evoked G_1_-phase arrest and antiproliferation. It is worth noting that, in addition to cyclin D1, the E3 ubiquitin ligase SKP2 is another key molecule to drive G_1_-to-S phase transition. SKP2 promotes G_1_ progression by inducing ubiquitination and proteasomal degradation of the cyclin-dependent kinase inhibitor p27^KIP1^, whose upregulation leads to G_1_-phase arrest [39]. Interestingly, we did observe SKP2 downregulation with paralleled p27^KIP1^ upregulation in colorectal cancer cells exposed to obatoclax (Supplementary Materials Figure S1A). However, ectopic SKP2 overexpression failed to abrogate obatoclax-induced suppression of clonogenicity, thus suggesting a dispensable role for the SKP2-p27^KIP1^ axis in this process (Supplementary Materials Figure S1B,C). Accordingly, cyclin D1 appears to be the prominent target with respect to the antiproliferative action of obatoclax.

Cyclin D1 overexpression is a common molecular alteration in various types of human cancers and has been identified in 55%~68% of human colorectal cancer cases [40,41]. Importantly, aberrant cyclin D1 overexpression is closely linked to cancer development, progression, poor prognosis, and chemoresistance, thus highlighting cyclin D1 as an attractive drug target [42,43]. Considering the labile nature of cyclin D1 protein, it is noteworthy that cyclin D1 accumulation in many human cancers is primarily attributed to impaired cyclin D1 degradation [30]. With these notions in mind, searching for agents that promote cyclin D1 degradation represents a promising strategy to discover novel cancer therapeutics [44,45]. To this end, our finding that obatoclax as a cyclin D1-ablating agent holds great possibilities for the inclusion of obatoclax in the treatment modalities targeting the cancer cells addicted to cyclin D1-conferred growth advantage.

Our mechanistic exploration revealed that obatoclax initiates cyclin D1 degradation through T286 phosphorylation-dependent or -independent mechanisms, depending on the nature of cell lines (Figure 4). In HT-29 cells, obatoclax appears to engage T286 phosphorylation-dependent cyclin D1 degradation (Figure 4A). However, it remains elusive as to the upstream kinases mediating obatoclax-induced T286 phosphorylation. GSK3β [31], ERK1/2 [32,33], p38^MAPK^ [34], and IKKα [46,47] are all known to phosphorylate T286 of cyclin D1. However, our data ruled out the involvement of GSK3β, ERK1/2, and p38^MAPK^ in obatoclax-elicited T286 phosphorylation, as evidenced by the findings that obatoclax provoked inhibition of GSK3β while barely activating ERK1/2 (Figure 4B), and that blockade of p38^MAPK^ activity failed to abolish obatoclax-induced cyclin D1 downregulation (Figure 4C). Therefore, future efforts are warranted to elucidate whether obatoclax activates IKKα to mediate T286 phosphorylation-dependent cyclin D1 degradation in HT-29 cells.

How obatoclax induces T286 phosphorylation-independent cyclin D1 degradation is currently unknown. One possible mechanism is through activation of Mirk/Dyrk1b, a kinase mediating T288 phosphorylation of cyclin D1 that leads to cyclin D1 degradation [48]. Alternatively, it is known that DNA damage lowers cyclin D1 expression through anaphase-promoting complex/cyclosome (APC/C)-mediated cyclin D1 degradation [49]. APC/C, an E3 ubiquitin ligase, initiates cyclin D1 degradation through direct binding to the N-terminal ExxL motif of cyclin D1. Given that obatoclax is capable of inducing DNA cleavage [50], it is also likely that obatoclax provokes genotoxic stress to engage APC/C-mediated cyclin D1 degradation.

As an inhibitor of multiple antiapoptotic BCL-2 proteins, obatoclax is generally regarded as an apoptosis inducer. Intriguingly, depletion of antiapoptotic BCL-2 proteins such as BCL-2, BCL-xL, and MCL-1 in colorectal cancer cells fail to suppress cell proliferation [51]. Therefore, the antiproliferative effect of obatoclax is apparently an apoptosis-independent process and represents another mechanism of action by which obatoclax elicits its anticancer activity. Aside from obatoclax, it is noteworthy that additional pan-BCL-2 inhibitors, such as gossypol and TW-37, also induce antiproliferation and downregulate cyclin D1 [52,53,54]. Accordingly, it would be interesting to further characterize how these pan-BCL-2 inhibitors downregulate cyclin D1, and whether antiproliferative action also applies to other BCL-2 inhibitors such as ABT-737, ABT-269, and ABT-199, whose targets of antiapoptotic BCL-2 proteins are more selective.

## 4. Materials and Methods

### 4.1. Chemicals

Obatoclax and MG-132 were obtained from AdooQ BioScience (Irvine, CA, USA) as 10 mM stock solution. Cycloheximide was purchased from Sigma-Aldrich (St. Louis, MO, USA).

### 4.2. Cell Culture

Human colorectal carcinoma cell lines HCT116 (ATTC^®^ No. CCL-247), HT-29 (ATTC^®^ No. HTB38), and LoVo (ATTC^®^ No. CCL-229) were grown at 37 °C and 5% CO_2_ in the culture media recommended by ATCC^®^ (American Type Cell Collection). All culture media, fetal bovine serum, and supplements were purchased from Invitrogen (Carlsbad, CA, USA).

### 4.3. Cell Proliferation Assay

HCT116, HT-29, and LoVo cells were seeded onto six-well plates at a density of 5 × 10^4^/well, followed by treatment with obatoclax (0, 50, 100, 200 nM). The numbers of cells at 24, 48, and 72 h after obatoclax treatment were harvested, resuspended, stained with trypan blue, and then counted using a Neubauer chamber.

### 4.4. Colony Formation Assay

Colony formation assays were performed using methods described in our previous report [55] to evaluate the effect of obatoclax on long-term cell proliferation. Briefly, cells (5 × 10^4^) were subject to stimulation by obatoclax (0, 100, 200 nM) for 24 h. Next, 2 × 10^2^ of obatoclax-treated cells were seeded on to six-well plates and allowed to grow in obatoclax-free culture media for 10~14 days to form colonies. Afterwards, colonies were rinsed twice by 1× PBS (phosphate buffered saline) and then stained with 1% crystal violet solution in 30% ethanol. The colonies composed of 50 or more cells were counted under microscopy.

### 4.5. Cell Cycle Analysis by Flow Cytometry

Flow cytometry was conducted as previously described [56]. In brief, cells (5 × 10^5^) were treated with obatoclax (0, 50, 100, 200 nM). Twenty-four hours after obatoclax treatment, cells were harvested, 1× ice-cold PBS-washed, and resuspended in 1 mL of 1× ice-cold PBS. Cells were then fixed by gradually adding 3 mL of ice-cold absolute ethanol and stored at −20 °C for 24 h. Next, cell pellets were collected by centrifugation, resuspended in 0.5 mL of 1× ice-cold PBS, and fully mixed with 0.5 mL of 0.1% Triton X-100 solution supplemented with 100 mg/mL of ribonuclease and 20 mg/mL of propidium iodide (PI) for 1 h-incubation at 37 °C in the dark. The levels of PI-DNA complexes-emitted fluorescence were monitored in a BD FACScan flow cytometer (Becton, Dickinson and Company, Franklin Lakes, NJ, USA).

### 4.6. Quantitative Real-Time Reverse Transcription-Polymerase Chain Reaction (RT-PCR)

Quantitative real-time reverse transcription-polymerase chain reaction (RT-PCR) was employed to determine the levels of *cyclin D1* mRNA in obatoclax-treated cells. Total RNA extraction, first-strand cDNA synthesis, and SYBR^®^ green-based real-time PCR were executed as reported previously [55]. The primer pairs used for *cyclin D1* real-time PCR include: forward, 5′-CTGTGCATCTACACCGACAAC-3′; and reverse, 5′-GTTCCACTTGAGCTTGTTCAC-3′. The levels of *cyclin D1* mRNA were normalized to that of TATA-binding protein (*TBP*). Final results are determined by the ratio of copy numbers of *cyclin D1* mRNA to the copy numbers of *TBP* mRNA and expressed as mean ± standard error of mean (SEM) from three independent experiments.

### 4.7. Immunoblot Analysis

Immunoblotting was performed in accordance to our established protocol [55]. If MG-132 treatment was required, 20 µM of MG132 was added 2 h before cell harvesting. Rabbit monoclonal antibodies against total cyclin D1, p-Cyclin D1 (T286), total GSK3β, p-GSK3β (S9), total ERK1/2, p-ERK1/2 (T202/Y204), total p38^MAPK^, p-p38^MAPK^ (T180/Y182), and hemagglutinin (HA) epitope were all purchased from Cell Signal Technology (Beverly, MA, USA). α-Tubulin antibody was purchased from GeneTex (Irvine, CA, USA). The signals were detected using an enhanced SuperSignal West Pico Chemiluminescence (Pierce, WA, USA).

### 4.8. Construction of the Retroviral Vector pBabe-Based Cyclin D1-Expressing Plasmid for Stable Clone Generation

The open reading frames (ORF) of human *cyclin D1* (Genbank accession No. NM_053056) was PCR-amplified from the first strand cDNA pools of HCT116 cells using the following primer pair: forward, 5′-GCCACCATGGAACACCAGCTCCTGTGCTGCGAAG-3′; and reverse, 5′-ACCGGTGATGTCCACGTCCCGCACGTC-3′. The PCR-amplified ORF was sequence confirmed and then subcloned to the retroviral vector pBabe.puro engineered to encode an in-frame C-terminal hemagglutinin (HA) epitope, generating pBabe-Cyclin D1-HA. Production of retroviral particles from pBabe-Cyclin D1-HA, subsequent cell infection, and puromycin selection for stable infectants were conducted according to our published protocol [56].

### 4.9. Cycloheximide Chase Analysis

Cycloheximide chase analysis was performed as described previously [56] to define the effect of obatoclax on the stability of cyclin D1 protein. Briefly, HCT116, HT-29, and LoVo cells (5 × 10^5^) were exposed to obatoclax (200 nM) for 18 h, followed by 6 h-treatment with cycloheximide (50 µg/mL) to stop de novo protein synthesis. Cyclin D1 levels at 0, 1, 3, and 6 h following cycloheximide co-treatment were then determined by immunoblotting.

### 4.10. Statistical Analysis

All data were represented as means ± SEM (standard error of the mean) from at least three individual experiments. Differences between groups were assessed for statistical significance using Student’s *t*-test. A *p*-value lower than 0.05 was regarded as the minimum criteria for statistical significance.

## 5. Conclusions

In this study, we for the first time elucidated the mechanism of action underlying obatoclax-mediated antiproliferative effect on human colorectal cancer cells. Specifically, induction of cyclin D1 proteasomal degradation accounts for obatoclax-elicited cyclin D1 downregulation, leading to G_1_-phase cell cycle arrest, and the consequent inhibition of cell proliferation. Our discovery that obatoclax targets cyclin D1 for degradation provides a novel insight into the anticancer mechanisms of obatoclax, but also identifies a potential application of obatoclax for the therapeutic ablation of cyclin D1 to treat the cancers addicted to aberrant cyclin D1 overexpression.

## Supplementary Materials

Supplementary materials can be found at www.mdpi.com/1422-0067/18/1/44/s1.
