# Supplementary material for: Obatoclax, a Pan-BCL-2 Inhibitor, Targets Cyclin D1 for Degradation to Induce Antiproliferation in Human Colorectal Carcinoma Cells
Source: Int J Mol Sci. 2016 Dec 27;18(1):44. doi: 10.3390/ijms18010044 (PMC5297679; doi:10.3390/ijms18010044)
Supplement: Supplementary file 1 [file ijms-18-00044-s001.pdf]

Supplementary Materials: Obatoclax, a Pan-BCL-2 Inhibitor, Targets Cyclin D1 for Degradation to Induce Antiproliferation in Human Colorectal Carcinoma Cells

Chi-Hung R. Or, Yachu Chang, Wei-Cheng Lin, Wee-Chyan Lee, Hong-Lin Su, Muk-Wing Cheung, Chang-Po Huang, Cheesang Ho and Chia-Che Chang

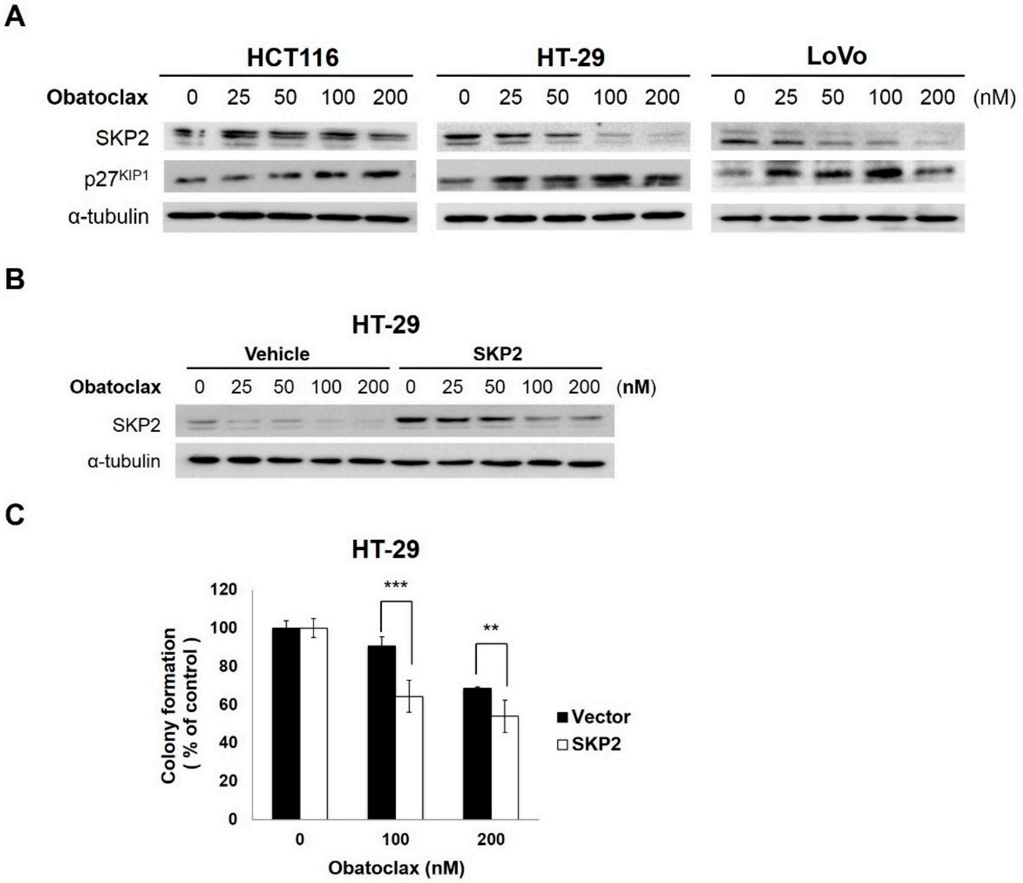

Figure S1. A dispensable role for the SKP2-p27^{KIP1} axis in the antiproliferative effect of obatoclax in human colorectal cancer cells. (**A**) Obatoclax downregulates SKP2 along witth p27^{KIP1} upregulation. Human colorectal carcinoma cell lines HCT116, HT-29 and LoVo were treated with grading doses of obatoclax for 24 h, followed by immunoblotting for the levels of SKP2 and p27^{KIP1}. α-Tubulin was used as the control for equal loading; (**B**) Generation of HT-29 clones with stable SKP2 overexpression. HT-29 cells were infected with pBabe.puro vector alone or with pBabe-SKP2 that expressed SKP2, followed by puromycin selection to establish SKP2 stable clones; (**C**) SKP2 overexpression cannot rescue HT-29 clonogenicity suppressed by obatoclax. Clonogenecity assay were performed in HT-29 stable clones of vector control or SKP2 treated with obatroclax (0~200 nM). **: $p < 0.01$; ***: $p < 0.001$.
